# Isolation, marginalisation and disempowerment – understanding how interactions with health providers can influence smoking cessation in pregnancy

**DOI:** 10.1186/s12884-022-04720-0

**Published:** 2022-05-10

**Authors:** Cherise Fletcher, Elizabeth Hoon, Angela Gialamas, Gustaaf Dekker, John Lynch, Lisa Smithers

**Affiliations:** 1 School of Public Health, Faculty of Health & Medical Sciences, University of Adelaide, Level 5, 50 Rundle Mall, Rundle Mall Plaza, Adelaide, SA 5005 Australia; 2 Discipline of General Practice, Faculty of Health & Medical Sciences, University of Adelaide, Adelaide, SA 5005 Australia; 3 The Robinson Research Institute, Norwich Centre, Ground Floor, 55 King William Road, North Adelaide, SA 5006 Australia; 4 Population Health Sciences, University of Bristol, Bristol, UK; 5 School of Health & Society, Faculty of Arts, Social Sciences & Humanities, University of Wollongong, Wollongong, NSW 2522 Australia; 6 The Illawarra Health and Medical Research Institute, University of Wollongong, Northfields Avenue, Wollongong, NSW 2522 Australia; 7 Obstetrics and Gynaecology Department, Lyell McEwin Hospital, University of Adelaide, Adelaide, Australia

**Keywords:** Tobacco smoking, Pregnancy, Smoking cessation, Socioeconomic position, Disadvantage

## Abstract

**Background:**

Maternal smoking during pregnancy can lead to serious adverse health outcomes for both women and their infants. While smoking in pregnancy has declined over time, it remains consistently higher in women with lower socioeconomic circumstances. Furthermore, fewer women in this group will successfully quit during pregnancy.

**Aim:**

This study explores the barriers to smoking cessation experienced by socially disadvantaged pregnant women and investigates how interactions with health providers can influence their smoking cessation journey.

**Methods:**

Women (either pregnant or birthed in the previous 10 years, who smoked or quit smoking in pregnancy) were recruited from a metropolitan public hospital antenatal clinic in South Australia and community organisations in surrounding suburbs. Seventeen women participated in qualitative semi-structured small focus groups or interviews. The focus groups and interviews were recorded, transcribed and thematically analysed.

**Findings:**

Four interconnected themes were identified: 1) smoking embedded in women’s challenging lives and pregnancies, 2) cyclic isolation and marginalisation, 3) feeling disempowered, and 4) autonomy and self-determination. Themes 3 and 4 are characterised as being two sides of a single coin in that they coexist simultaneously and are inseparable. A key finding is a strong unanimous desire for smoking cessation in pregnancy but women felt they did not have the necessary support from health providers or confidence and self-efficacy to be successful.

**Conclusion:**

Women would like improvements to antenatal care that increase health practitioners’ understanding of the social and contextual healthcare barriers faced by women who smoke in pregnancy. They seek improved interventions from health providers to make informed choices about smoking cessation and would like women-centred care. Women feel that with greater support, more options for cessation strategies and consistency and encouragement from health providers they could be more successful at antenatal smoking cessation. If such changes were made, then South Australian practice could align more with best practice international guidelines for addressing smoking cessation in pregnancy, and potentially improve outcomes for women and their children.

**Supplementary Information:**

The online version contains supplementary material available at 10.1186/s12884-022-04720-0.

## Introduction

“*… yes, there is a lot of pressure on quitting smoking, but there’s not a lot of support on how to do it.”*

*Woman who smoked during pregnancy FG4.*

It has been well established that smoking in pregnancy can result in poor maternal and infant outcomes. Smoking can increase the rate of miscarriage, stillbirth, preterm birth, fetal growth restriction and placental abnormalities [1–5]. In addition to pregnancy complications, children born to mothers who smoke in pregnancy exhibit increased respiratory dysfunction [6, 7], impaired cognitive development [8–10], behavioural difficulties [11, 12], obesity [13, 14] and an increased risk of Sudden Infant Death Syndrome (SIDS) [5], as well as future nicotine dependence [1, 2, 15]. In the UK, it is estimated that children born to mothers who smoke cost the National Health Service (NHS) the equivalent of between £12 million and £24million (~$AUD21 and $AUD43) just in their first year of life, directly related to smoking in pregnancy [16]. Evidence does, however, suggest that smoking cessation in pregnancy prior to 20 weeks gestational age can eliminate the excess risk attributable to smoking [2, 17, 18].

In 2018, the Australian Institute of Health and Welfare (AIHW) reported that 9.6% of women who gave birth smoked in pregnancy [19]. However, tobacco smoking in pregnancy is higher among women living in socioeconomically disadvantaged circumstances [19–23]. The AIHW in 2018 confirmed this finding with a reported 17% of women living in low socioeconomic areas smoking in pregnancy [19]. Qualitative systematic reviews by Flemming et al. 2013 and 2015 detailed the experiences of disadvantaged pregnant smokers and the barriers they face with cessation [20, 24]. Some of the barriers to smoking cessation in pregnancy included the embeddedness of smoking in women’s lives, women’s psychological well-being, relationships with significant others, and women’s appraisal of the risk of smoking [20, 24]. The reviews concluded that social disadvantage influenced by chronic stressors, cultural practices, limited economic resources and unsupportive social relationships effect women’s ability to attempt, or be successful at smoking cessation in pregnancy [20, 24]. While the reviews offer insight into how the circumstances of women’s lives influence their smoking behaviour in pregnancy, just how interactions with antenatal healthcare providers can affect women’s experience with reference to quitting, has not been explored.

The current South Australian (SA) Perinatal Practice Guidelines (SA PPGs) recommend that health providers complete an initial comprehensive screening of smoking and nicotine dependence for every woman antenatally, including discussion about understanding the risks of smoking in pregnancy, the benefits of smoking cessation and options for support of cessation incorporating nicotine replacement therapy availability [25]. The SA PPGs further recommend that routine support (overview of tobacco use, cessation supports and NRT) and follow up at each antenatal appointment is offered with health providers and that this is documented in the clinical case notes. They define 5A’s (Ask, Advise, Assess, Assist, Ask Again), a brief intervention approach to use as a mnemonic for health providers in addressing each component of the comprehensive screening and follow up with women. The SA PPGs recommend adopting the 5A’s at a minimum when discussing smoking cessation with pregnant women [25]. In practice, health providers complete a brief intervention with the 5A’s at the initial triage (first hospital visit) appointment with women at approximately 10 weeks gestation. This intervention is limited to enquiring about smoking status, assessing nicotine dependence and offering women who smoke a referral to the telephone counselling service, Quitline, if they indicate they would like to quit smoking. Women are informed of this service by midwives or obstetricians that provide antenatal care in the hospital antenatal service. Health providers may submit a referral to Quitline on behalf of the woman so they can be contacted by the service, or women can contact Quitline directly via a phone number provided by the same health providers in the hospital service. In practice, other than Quitline, women are unlikely to be offered any other support or advice about cessation or NRT use by health providers in the hospital service [26]. The SA PPGs relating to tobacco use in pregnancy were last updated in 2013 and currently state that there is a lack of evidence of the safety of NRT in pregnancy, and that women should only consider NRT in certain circumstances [25].

In Australia it is estimated that only 4% of women who smoke will cease smoking in pregnancy [7], and it is estimated that this is lower among women who live in disadvantaged circumstances [27]. If women engage with the Quitline service, then ongoing telephone counselling is available. Women will be informed by the telephone counsellors that they can discuss NRT use with their general practitioner (GP). This approach to smoking cessation in pregnancy advice has not changed in 15 years in SA. This current approach to antenatal smoking cessation may not be supportive enough for women’s quit attempts. This has been reinforced in the literature by Bowden et al 2010 [28], whose study conducted in metropolitan Adelaide followed integration of the 5A’s in clinical practice. Approximately 90% of staff incorporated the 5A’s into clinical practice and ~ 80% of pregnancy records had a completed Smoke-Free Assessment & Intervention Form (SFA&IF) which covers the 5A’s in detail [28]. However, only 5% of pregnant women sustained quitting to 5 months and only 50% of current smokers were provided with smoking cessation assistance and support by antenatal care providers [28]. Given these findings and that smoking cessation in pregnancy has not increased in the previous 10 years, considering a broader approach to smoking cessation with an understanding of women’s circumstances and needs could be beneficial. It has been suggested that health providers encounter considerable barriers antenatally addressing smoking cessation including time constraints, lack of training or skill, administrative barriers and lack of acceptable interventions [29–31]. The aim of this study is to further explore and understand the barriers to smoking cessation experienced by pregnant women who live in a socioeconomically disadvantaged area and examine how the interplay with primary antenatal care providers influence women’s smoking or cessation journey.

## Participants, Ethics & Methods

### Theoretical perspective

This research project was motivated by discussions with a community reference group formed in northern Adelaide. This reference group was established explicitly to promote a program of work focussed on smoking cessation in pregnancy. The reference group endorsed a qualitative study conducted with the aim of understanding the breadth and depth of participant experiences. An interpretive phenomenological approach was used to elicit common themes of participants’ lived experiences of either smoking or quitting in pregnancy. This approach describes the meaning of their experiences in terms of what was experienced and how it was experienced [32]. By examining subjective lived experiences and meanings it is possible to develop new understandings and appreciations to inform or re-orient how we interpret those experiences [32, 33]. It is through this lens, prioritising the subjective perspectives of women accessing antenatal care in the South Australian healthcare system, that we aim to understand their experiences around smoking and cessation in pregnancy. The researchers acknowledge a constructionist epistemological position where meaning arises in and out of our engagement with the realities in our world and is advantageous in generating contextual understanding of a defined topic, for example, smoking and/or cessation in pregnancy [34]. This position also assumes that different individuals will construct meaning of the same phenomena in different ways and how that individual engages with and understands their world is established from their cultural, historical and social perspectives [34].

This study was approved by the Central Adelaide Local Health Network (CALHN) Human Research Ethics Committee (HREC), approval number HREC/19/CALHN/119 Q20190304. All participants were provided with a participant information sheet and provided written informed consent prior to participating.

### Sample and recruitment

Women were recruited from a single metropolitan public hospital antenatal clinic in South Australia and surrounding suburbs at community centres, community organisations, playgroups and kindergartens. All recruitment locations were situated in regions within the lowest quintile Index of Relative Socio-economic Advantage and Disadvantage (IRSAD) of the Socio-Economic Indexes for Areas (SEIFA) as indicated by the Australian Bureau of Statistics 2016 census data. The SEIFA IRSAD is an area-based measure of socio-economic advantage and disadvantage and includes variables related to both socio-economic advantage and disadvantage such as education, income, occupation and housing [35]. Women recruited were aged 18 years or over, could communicate proficiently in English, were pregnant or had given birth to children in the northern suburbs of Adelaide (an area of low socioeconomic strata) in the previous 10 years and either smoked cigarettes or quit smoking cigarettes in pregnancy. In the hospital, health professionals would identify eligible women from their antenatal medical case notes and refer them to a researcher to discuss the project. In the community, the researcher (CF) would talk to women (individually) attending community hubs, playgroups or kindergartens about the project and determine if they were eligible to participate. After discussion with the researcher (CF) about the project, contact details were taken, women were given information and up to a week to decide if they would like to participate. The researcher CF would then phone women and arrange a time to attend a focus group or interview if they agreed to participate. On attendance at a focus group or interview, women were asked to sign a consent form. Initially, the sampling strategy was to recruit only women who were currently pregnant from the antenatal clinic in a single specific northern public hospital. However, in response to low recruitment levels and researchers unable to enter the health service during the SARS-CoV-19 pandemic, the criteria were expanded to include women who had been pregnant at any point in the previous 10 years from multiple community organisations surrounding the hospital.

### Data collection and analysis

Women who provided informed consent, participated in qualitative semi-structured face-to-face small group interviews (*n* = 2–4) or 1-on-1 in-depth interviews (face-to-face or by phone) between February 2020 and September 2020. The intent was to run focus groups with four to six women. However, co-ordinating this with women, as well as addressing COVID-19 restrictions proved challenging and meant that the number of women present depended on their availability on the day. Discussions were held at local community centres with two researchers facilitating and observing the session (CF and EH). Women were asked to complete a short-written questionnaire on arrival to collect demographic information (age, ethnicity, highest level of qualification, employment status, number of pregnancies, smoking history and age of smoking commencement) and previous attempts at smoking cessation in pregnancy. In accordance with ethics approval and maintaining the confidentiality of women, demographic data where *n* < 5 will not be presented. Focus groups were approximately 2 h duration and interviews between 20 to 50 minutes. Topics for discussion at focus groups and interviews are in Additional file 1. Briefly, discussion included women’s experience, reflection and emotion around smoking in pregnancy, and their antenatal experience with health providers on topics such as risk, cessation strategies and Quitline. Focus groups and interviews were established at local community spaces with creche services available for focus groups. Transport was provided, if required. Women were provided with a $50 gift card in remuneration for their time and expertise. At the end of each focus group both researchers debriefed on key learnings and reflections, a key component of reflective research. CF also completed reflective field notes of the data collection episode.

Recruitment and hence focus groups/interviews continued until thematic saturation was reached. All discussions were audio recorded and transcribed verbatim. Transcripts were assessed against the original audio recording and reviewed. Researchers familiarised themselves with the material and the data was coded and thematically analysed as described by Braun and Clarke [36]. Data was coded inductively, that is, without using a pre-conceived conceptualisation or existing theory. Related and similar codes were collated into overall themes. Data analysis was predominately done by a single researcher (CF); in the early stages of analysis CF had discussions with the experienced qualitative researcher (EH) who had attended each focus group. As analysis progressed, there was ongoing collaborative discussion of all themes and iterative revisions of coding by all researchers. NVivo 12 qualitative analysis software (QSR International) was used for coding, analysing and managing data.

## Findings

Recruitment for this project was very difficult. Women were very reluctant to engage on the topic. State-wide COVID-19 lockdowns and restrictions also hampered recruitment with researchers unable to recruit in the hospital or community. Between February 2020 and September 2020 there were 50 eligible women who spoke with a researcher (CF or a member of the broader research group), either in the hospital antenatal clinic or community organisations where contact details were exchanged. Many women (*n* = 25) who met with researchers either declined to participate, or were uncontactable in the weeks following that meeting. This was mainly due to women’s reluctance to engage on the topic, due to stigma and fear of judgement. In some cases (*n* = 8), women expressed an intent to participate, but despite several opportunities being offered, current commitments meant that they were unable to attend a focus group or interview. All women were offered the opportunity to do a phone interview. There were 17 women who consented to be involved in the project and were involved in a focus group or interview. Three focus groups with 3–4 participants each, two small group interviews each with 2 participants and three 1-on-1 interviews were conducted. It was observed that women involved in all focus groups and interviews, regardless of the age of their youngest child, had strong emotive recollections of their experience of smoking in pregnancy and the antenatal care they received related to smoking.

Four interconnected themes were identified as impacting the smoking cessation journey for women in pregnancy during antenatal care. The first, ‘smoking embedded in women’s challenging lives and pregnancies’ forms the foundation on which the following three themes are layered. The second, ‘cyclic isolation and marginalisation’ is an extension of the first and exists because of the embedded nature of smoking in their lives. The final two themes are characterised as being two sides of a single coin in that they coexist simultaneously and are inseparable. They are intensified by the existence of the first two themes and represent women ‘feeling disempowered’ in their interactions with healthcare providers and desiring ‘autonomy and self-determination’ in their smoking cessation journey.

### Participant characteristics

A total of 17 women were included in the study. Characteristics of participants collected at the time of focus group or interview are detailed in Table 1. The mean age of women was 33 years (range 24–43 years). The majority of women (*n* = 12) identified as Caucasian. The mean age of cigarette smoking commencement was 14 years, with the youngest age of smoking commencement at age nine. Most of the women (*n* = 12) had either completed high school or vocational training. At the time of their focus group or interview, the majority of women (*n* = 12) were in a carer/home duty role or were unemployed. All women resided in areas within the lowest IRSAD quintile. This was reflected by the majority of women (> 70%) holding an Australian Healthcare card, where the Australian government sets eligibility at a weekly household income below $1130. Approximately one third of women were pregnant during participation in the project. Over 80% of women had been pregnant at least twice, with 6 having experienced 5 or more pregnancies at their inclusion in the study. Of the women who participated, fewer than 30% were able to successfully quit smoking during all of their pregnancies, however, most of the women who had effectively quit in pregnancy (> 70%), had begun smoking again postnatally and were smoking at the time of participating in the study. Overall, a considerable number of participants had attempted to quit smoking in some or all of their pregnancies (*n* = 12) but had found quitting unsustainable. Women who were not pregnant at interview had a youngest child ranging in age from 4 months to 8 years.

**Table 1:** Demographic characteristics of 17 women in northern Adelaide, SA

Characteristic	Mean (range)	N
**Age (years)**	33 (24–43)	17
**Age started smoking (years)**	14 (9–18)	17
**Number of pregnancies**		
1–4		11
≥ 5		6
**Ethnicity**		
Caucasian		12
Other^a^		5
**Education**		
Did not complete high school		5
Completed high school or vocational training		12
Employment		
Carer/home duties or unemployed		12
Studying or paid work		5
**Attempted to quit smoking in pregnancy**		
Yes		12
No		5

^a^ Combines Asian, Pacific Islanders, Aboriginal and Torres Strait Islanders

### Smoking embedded in women’s challenging lives and pregnancies

For the women interviewed, smoking cigarettes is deeply embedded in their lives. Women described using smoking to manage negative emotions in their lives, including stress, anxiety, anger, guilt and frustration. In fact, women often referred to the chaos in their lives and how smoking played an extensive role in helping them cope.

*‘ … because I probably smoked the heaviest towards the end of my pregnancy, but that’s just because I had so many emotions going on at the time that it was my only – I felt it was my only coping mechanism … ’  FG1.*

*‘ … when you live in our community, and you’ve had an upbringing like mine, you’ve gone through the system, you’ve had all that shit. Like a cigarette sometimes is like your sanity.’ FG3.*

They also described using smoking as a celebratory or reward activity to have time to themselves for relaxation, mindfulness and reflection.

*‘That’s my five minutes, and with five kids at home I need that five minutes.’ Int3.*

Women further described associative behaviours, habit and boredom as factors in perpetuating their smoking.

*‘You don’t even think about it. Before you knew it, you’re grabbing one, and you’re going outside … ’ FG4.*

In pregnancy, smoking cessation was often considered a low priority while dealing with everything else in their lives. Women specifically mentioned issues such as domestic violence, single parenting, child protective services, family breakdown, pregnancy hormonal fluctuations, ‘mum guilt’ and mental health problems.

Furthermore, women reflected that the acceptability of using smoking as a coping strategy has shifted over time. Where smoking was once an acceptable, resourceful way of managing, now, the dominant health discourse dictates complete abstinence from smoking. However, women felt that when smoking has been embedded in their lives for so long and is their *only* coping mechanism, they had little choice but continue to smoke.

*‘ … When I started – 14 [it] was 2000, so although there wasn’t the smoking culture that there was when my parents were younger, it still wasn’t such a big deal, and you would hear about kids at school going to the back of the fence and having a smoke, and stuff like that.’ Int3.*

*‘[my partner was away and people were laughing at me] I smoked more and more and more, and it was just harder to sleep, and it was harder to do a lot of things, and I fell right down, and … I was in that zone where I couldn’t do nothing … ’ FG4.*

While these findings have been documented in previous studies, it is fundamental that it is represented here to form a situated understanding of the participants’ lives as these perspectives relate to the further themes generated in this analysis.

### Cyclic isolation and marginalisation in antenatal care

In pregnancy, women reported persistently experiencing alienation, judgment, intimidation and stigma around smoking from the general community, health service providers and sometimes their own smoking social circle. Women discussed concealing their smoking out of feelings of shame and guilt. They further discussed how this concealment contributed to exclusion and segregation from their social group and the general community, contributing to feelings of isolation.

*‘So it’s feeling that alone, and then you seclude yourself from social gatherings, because you’re like, I don’t need that person judging me.’ FG1.*

*‘ … And then like when I started showing and stuff, in public I would, you know, tuck myself away somewhere, because I didn’t want people making judgments on me, because I actually was trying [to quit].’ FG2.*

Women who decided to quit smoking in pregnancy also struggled with isolation. Several women reported that they experienced persistent peer pressure from their families and social group to continue smoking when the group was together, and they found that exposure to second-hand smoke (SHS) was a constant temptation to smoke. This meant that women found it essential to isolate if they wanted to remain abstinent.

*‘You’re having like a mental breakdown, because you’re like pregnant, and your emotions are like all over the place, and they’re like, “Just come have a smoke.” “I don’t want to smoke.” “Just come and have a smoke, you will feel better.” ﻿’ FG3.*

*‘It’s really hard. Like if you’ve got someone smoking – well, I’ve got the patch on, and I had to just go to my partner’s work to drop off smokes to him and bring him food and stuff, and then because he had a smoke I ended up having a few drags of it.’ Int1.*

In the antenatal healthcare setting, women reported that they were often apprehensive of having conversations about smoking in pregnancy due to perceived persistent judgment and stigma from healthcare professionals. They perceived a lack of understanding about the role of smoking in their lives from health professionals and that the discussion at their first appointment about quitting smoking is simply ‘a box to be ticked’ (FG1 & FG3). They felt this especially as there is rarely any follow-up about the topic at subsequent appointments, despite this being encouraged by the SA PPGs. Women did not feel encouraged to seek support about smoking or quitting in pregnancy from the healthcare system and this consequently contributed to and perpetuated the cycle of isolation.

*‘It’s, “Do you want go quit?” “Yes.” “Here’s the Quitline number.” That’s all I ever got.’ FG1.*

*‘I was in low care with her, and I just felt with the low risk that I didn’t get a lot of support in anything, to be honest with you. Yes. I was on my own. That’s how it kind of felt … ’ FG4.*

*‘﻿You feel like you’re just a number and then you’re on the conveyor belt, just going through the system.’ FG3.*

All women remembered discussing Quitline with antenatal health providers and were aware of the telephone counselling service. However, the majority did not recall health providers offering to submit a referral to the service on their behalf, only that they could contact them independently via the phone number health providers supplied them with. Women expressed very negative attitudes towards Quitline and most had a pre-conceived idea that the service was ‘useless’.

*‘That’s not going to help you quit smoking … *I*t was stupid.’ FG1.*

Then, when women felt they were expected to engage unassisted with Quitline (even if they thought the service was unhelpful), this contributed further to feelings of isolation.

*‘ … like it was, “There’s the number. There you go. You can handle it. You can do it yourself.”’ FG1.*

*‘But even if you ask you’re just given a brochure, and that’s it. There was nothing – there was no, you know, more talking about. It was just, “Here you go.”’ FG2.*

*‘She [the midwife] knew that I struggled with giving up, and that there has been an increase in my smoking … she has asked me if I wanted to ring Quitline, and that’s it. You know, that’s obviously the only option that she has available for me … ’ FG5.*

Furthermore, women that had experienced antenatal care several times over the previous 15–20 years due to several pregnancies (*n* = 5), criticised that there had been no change in the way smoking was approached or discussed in antenatal care, only that they perceived more judgement from health professionals. As stated earlier, twelve women reported quit attempts in pregnancy, but only four of these women attempted to engage with Quitline. Of the four that engaged with Quitline, three were women who engaged with Quitline while pregnant with their elder children (children now aged between 15 and 18), found the service unhelpful and then declined to contact them for future pregnancies.

*‘I’ve never been a fan of Quitline. I’ve spoken to them multiple times … they just throw numbers and maybe sometimes throw a free trial of product out.’ Int3 (multiple pregnancies).*

This impersonal approach in antenatal care to address smoking in pregnancy contributes to women feeling socially marginalised. Women also felt that pregnant smokers were unfairly classified or pigeonholed by the health system. That is, they felt that certain assumptions are made about women who smoke in pregnancy, for example that they are impoverished and uneducated.

*‘ … but I also didn’t want to be classed as a feral … You know … “Trashy mum,” or, you know, anything like that … ’ FG4.*

*‘Or thinking that you don’t care about your health or your kid’s health or that you deserve whatever happens to you … and it’s not that you don’t care or don’t know, and then that makes you upset, which makes you go and have a smoke.’ Int3.*

Then, despite the recommendation in guidelines to offer several cessation support options, when women are offered the single cessation option, Quitline, women found this patronising and condescending. Women would like to be seen as individuals, rather than as a collective group that are all given the same solution.

*‘ … it seems condescending … and it just ticks me off.’ Int3.*

Additionally, women that attempted smoking cessation in pregnancy did not seek assistance from general practitioners (GPs) or pharmacists outside the antenatal hospital system with cessation methods or further support. This extended and perpetuated the cycle of isolation women experienced related to healthcare providers. Furthermore, women received no information from hospital-based health providers, Quitline, GPs or pharmacists (due to lack of engagement) about accessing or using NRT in pregnancy. In fact, the majority of women interviewed were unaware that NRT is safe to use in pregnancy.

*‘ … as well, you can’t even really use anything to quit when you’re pregnant either.’ Int1.*

The few women who had quit smoking in pregnancy reflected that they did so independently and did not recall receiving any support from healthcare services or providers.

### Different faces of the same coin. Side 1 – feeling disempowered

As a result of isolation and marginalisation, women felt disempowered to quit smoking in pregnancy. They reported that they were provided with limited information to make informed choices about smoking in pregnancy. This is not consistent with the SA PPG recommendations that encourage health providers to discuss with women the ‘potential harmful effects of smoking on the fetus’ [25]. Women could reliably attest that ‘smoking in pregnancy is bad’ and that the baby could have low birth weight, but they could not be specific about any other smoking related adverse health outcomes.

*‘I don’t know a lot of the risks, but I know obviously it’s not good for the baby.’ Int1.*

*‘But, I mean, they do warn you about not smoking. They just don’t tell you why … ’ FG1.*

*‘ … it’s never, “You should quit smoking. There’s risks involved. These are the risks.” It’s – it never gets elaborated. It’s just they’re sort of like, “Uh, all right. I’ve done that. Tick that off. I told you to quit smoking. That’s all I really – that’s all – that’s part of my job and it’s part of the system.”’ FG1.*

*‘It’s raping us of our knowledge really, and if they’re going to accuse us of causing smaller children and … possibly killing our babies due to smoking cigarettes, but not giving us statistics or … yes.’ FG5.*

For the few women who did describe being provided with information about risk or cessation strategies, further disempowerment occurred with inconsistent messaging from health providers. Women found it challenging to distinguish credible information from out-dated or incorrect information due to the discordance of advice from health providers on the topics. This had an impact on the professional clinical care relationship with women expressing less confidence in their healthcare providers.

*‘Having that different personalities. You have one [midwife] – like one could be really laid back about it, like, “I understand that it’s hard,” and then you’ve got another one that’s like, “You should be quitting.”’ FG1.*

*‘I think a lot of – there’s a lot of inconsistencies from doctors and chemists and the Quitline, and that makes it hard … ’ Int3.*

The majority of women expressed strong reluctance to engage with Quitline and they felt disempowered to quit given this reluctance. Those with experience of Quitline expressed that they found the service unhelpful. Women felt a sense of futility to quit as they assumed (and in some cases been advised by Quitline) their only quitting options were cold turkey and the harm reduction approach of reducing the number of cigarettes smoked in a day.

*‘I called up, and I hung up straight away … Because I didn’t want to call somebody and talk about my smoking over the phone. I was like, “Look at this bullshit. Like what are they going to do for me? Nothing.”’ FG5.*

*‘I’ve never been a fan of Quitline … I spoke to Quitline, and they said, “There’s nothing. You can’t use anything in pregnancy. It’s not safe. You just have to put down the smokes and walk away.”’ Int3.*

*‘I have tried like cold turkey, but that doesn’t work. Doesn’t work for me.’ FG4.*

Several women also expressed futility in smoking cessation, stating that they felt smoking was a choice where they had insufficient willpower and self-efficacy to successfully quit.

*‘ … if I could give up and have it be so easy, yes, I would give up and have it be so easy, but I still do choose to smoke. It is something that I enable myself to do. So there’s where the guilt comes from, because it is a choice.’ FG5*

*‘Like with my first pregnancy, I was a lot stronger than what I am now. I don’t have as much strength as what I did have then, and if I did have the strength and the support system, you know … ’ FG5.*

Even with disempowerment and futility, women still expressed a strong desire to be valued and respected and this presented itself in the form of self-soothing statements using “lay epidemiology” [37].

*‘So I wasn’t too worried. My mother smoked through – when she was pregnant with me. I was prem, but it wasn’t from her smoking, because she cut straight back.’ Int2.*

*‘See, I don’t know, because, like I said, a lot of people I know smoked in their pregnancies, and their babies were fine.’ Int1.*

### Different faces of the same coin. Side 2 – autonomy and self-determination

Despite feeling disempowered, isolated and marginalised, women also expressed a desire to want to quit smoking in pregnancy. This desire was driven principally by their moral obligation to care for and protect their baby. Women however continually expressed that achieving abstinence in pregnancy was unattainable when there was no professional or social support, support which is encouraged by current SA PPGs.

*‘ … Because, at the end of the day, every mother wants their baby to be as healthy as possible.’ Int2.*

*‘ … so it’s just not the right thing to do, it’s not the best start for the baby … ’ FG4.*

*‘ … yes, there is a lot of pressure on quitting smoking, but there’s not a lot of support on how to do it.’ FG4.*

Women identified that they would like to feel safe and supported having conversations antenatally about smoking and quitting in pregnancy. They reported that they want more information about risk and cessation strategies to make informed decisions, as well as to form trusted relationships with healthcare providers who offer consistent information. These considerations are again all encouraged and promoted by the current SA PPGs. The few women involved in this study who had experienced continuity of care identified that having rapport and an established relationship with their healthcare provider positively impacted their ability to have challenging discussions around smoking behaviour change. Women who did not have antenatal continuity of care coveted this experience and felt it would make quitting easier.

*‘ … with my first pregnancy, because I did have that great rapport with my midwife … – I think communication works really well when you’ve got a good connection, and I think that that’s the key to sharing information … like if you’ve got a stranger who tries to say the same thing you might be like, “I don’t know,” … I think it’s definitely the relationships that you’re able to have with your professionals that could help with that … ’ FG2.*

*‘You probably would [have a better relationship with the same midwife], but I never had – I never got to have the same midwife, because it was always a different … ’ FG3.*

Women also expressed a strong interest in engaging with peer-to-peer support for women who smoke or quit in pregnancy. Some women thought this would be good in a face-to-face group facilitated by a health practitioner, while others preferred the idea of a virtual/online group through social media platforms.

*‘Like even just a Facebook group of people that are sort of all in the same boat could be helpful, where you can talk to people but not know them.’ Int3.*

*‘So I think having a group like that with like-minded women that are going through the same thing, I think would be really good and a really good support system.’ FG1.*

By having more information, improved relationships with health professionals and peer support, women ultimately expressed a desire to exercise autonomy and choice over their quit journey. Furthermore, given the complexity of their lives, and the embedded nature of smoking in those lives, women identified the need to support personal agency and self-determination to decide what works best in their own circumstance rather than the current ‘one size fits all’ approach with being offered Quitline.

*‘If there’s someone who’s wanting to give up … [Instead of Quitline it could be:] “Look, we will give you a few different options to try” … give us more options, more welcoming options.’ FG5.*

*‘ … because there’s not one way for everybody. There’s a 100 ways for 100 different people, you know … ’ FG2.*

## Discussion

The present study confirms similar findings that smoking is a deeply embedded and personal practice for women, but provides new insights into how interaction with health providers can generate additional barriers that coalesce with social barriers, to limit successful smoking cessation in pregnancy. The authors do acknowledge that broadening the eligibility criteria to include women who had birthed ‘up to 10 years previously’ is a limitation of the study. However, inclusion of women who had birthed over an extended timeframe did highlight that the approach to smoking cessation in SA antenatal clinics has not substantially changed in many years, and that therefore reflections from women who birthed children up to 10 years ago or multiple times over many years (up to 20) remain relevant to this research question. Systematic reviews published in 2013 and 2014 [20, 24], as well as a more recent Australian publication [38], emphasize the central role smoking plays in women’s lives as a coping strategy. Despite women expressing the necessity for smoking in their lives, in pregnancy the feelings of shame, guilt and self-loathing are strong, and drive the constant internal struggle between addiction and desire to quit. Furthermore, quitting smoking during pregnancy here and in other work [24, 39] has been considered by women to be of low importance in the hierarchy of difficult problems in their lives.

Although women described barriers in pregnancy that prevent them from quitting, a key finding here is a strong, unanimous desire from women to be non-smokers in pregnancy. Due to women’s reluctance to discuss smoking during antenatal care, health providers perceive women who smoke in pregnancy as having low motivation to quit [26, 40]. However, for women here, it is the overwhelming feelings of shame, guilt and isolation, coupled with the judgment they feel from healthcare providers that contributed to their reluctance to discuss the issue. This has been seen in previous work and has been seen to coincide with women under-reporting their smoking behaviour [41–46].

Without the relevant information or appropriate support from the health service or their social circle for smoking cessation, women who smoke in pregnancy feel isolated and marginalised. Isolation is a derivative of behaviour identified in previous work, where women modify their smoking behaviour to smoke in morally safe spaces, that is, in secret or hiding [38, 46, 47]. Although women are hesitant about conversations with health providers, the ‘tick the box’, detached, superficial approach at their initial appointment – only offering Quitline if there was an interest in quitting and lack of follow-up – also contributed to isolation, marginalisation, and a minimisation of the importance of smoking cessation in pregnancy. In line with a 2008 report [48] the women participating in this study would identify with the archetype of already being marginalised Australians, thus the feelings of isolation and marginalisation are amplified when reflecting on their interactions with the health system. There have been five domains of disadvantage identified that contribute to marginalisation in Australia: social stigmatisation, early-life disadvantage, financial hardship, poor health and social isolation [48]. Reducing smoking in Australia is a public health priority; however, policy determined by government aimed at achieving this may contribute to an already marginalised group feeling further undervalued and disenfranchised. Anti-smoking public health approaches can contribute to exacerbating four of the five domains of marginalisation in Australia by increasing financial hardship, poor health, social stigmatisation and social isolation for this group of women. Furthermore, the expectation that women engage with Quitline independently and in parallel to their antenatal care reduces the capacity for a supportive multidisciplinary approach to smoking cessation in pregnancy and thus contributes to social isolation.

Given women report limited conversations with health providers or Quitline about the risk of smoking in pregnancy, or smoking cessation options available to use in pregnancy, it follows that they feel unable to make informed decisions about their smoking behaviour. Previous work has indicated that women were aware of the risks to themselves and their baby from smoking in pregnancy [38, 41]; however, risk was considered on a population level as a whole and was therefore a disembodied risk and not a personal one [20]. Women have also engaged in ‘lay epidemiology’ to draw their own conclusions about the relative risk of smoking in pregnancy [20, 38, 49]. In these examples, women used anecdotal and observational evidence to refute the public health messaging of risk [20, 38, 49]. Women would use their evidence to rationalise and defend their continuation of smoking. This is in contrast to women in this study using lay epidemiology as a method of self-soothing in the face of disempowerment and futility. While both are seen to refute public health messaging, the underpinning sentiment differs. The former is from a defiant and justifying position, whereas women here felt they lacked will power and self-efficacy, and therefore used lay epidemiology to reassure and alleviate guilt.

A further challenge for women negotiating risk and cessation strategies is observed when there is inconsistent messaging among health providers. In Hansen et al. [38] women who smoked in pregnancy used inconsistent messaging from health providers as a way to bias their personal anecdotal evidence pool with lay epidemiology. However, the findings here indicate that inconsistent messaging from health providers lead to women feeling disempowered and weakens the professional relationship women have with those health providers. Women here and in other studies are eager for consistent information about risk and cessation strategies from non-judgemental health providers that consider their individual needs [40, 41, 50]. Conversely, health providers are concerned that if they force discussion about smoking cessation in pregnancy that this will result in non-attendance at antenatal care [26, 39, 44]. Also, there has been discourse among women and health providers about where the onus of responsibility sits with regard to smoking cessation in pregnancy. Reports have indicated that women feel that smoking cessation in pregnancy are individual decisions and the responsibility to quit is entirely theirs. As such, women do not want health providers interfering with their decision making and prefer the support of friends, family and other pregnant women [40, 44]. While the women interviewed here recognise that quitting is an individual decision, the disempowerment, marginalisation and futility they feel from interaction with the health service manifests in a sense of abandonment and neglect. Furthermore, due to the propensity of women using lay epidemiology as a valid form of evidence to either refute scientific findings or self-soothe (especially online [49]), it is vital that health providers are a reliable and consistent source of information. The findings here and elsewhere [20, 40, 41] suggest that communication from health providers around risk and cessation strategies could be an area of improvement to increase smoking cessation rates in pregnancy. Best practice according to the SA PPGs promote communication from health providers around risk, ongoing support and the discussion of smoking cessation strategies. However, the work here provides evidence that there is a disconnect between recommended best practice and actual clinical practice. It has been proposed that the limitations of health providers in antenatal care include their lack of training, knowledge and confidence in information delivery, perceived time restrictions and systems that do not support implementation and monitoring of smoking cessation in pregnancy [39, 51, 52]. In South Australia, health providers may also assume that if women are sincere about quitting, they will obtain information about risk and cessation strategies from Quitline (telephone counselling) or online sources. However, given women are so reluctant to engage with Quitline, an important opportunity is missed in antenatal care to provide women with consistent, accurate information and support. A 2019 Cochrane review indicated that telephone counselling for smoking cessation increased quit rates for participants that sought multiple sessions of proactive counselling [53], including in pregnancy [54]. Increased cessation rates were also observed in evaluations of the Australian (Victoria and South Australia services) Quitline. Despite the positive impact telephone counselling can have on smoking cessation, Gamble et al. [41] found that women wanted to contact Quitline when *they* needed assistance, not routinely at scheduled times [41]. This was consistent with women in the current study who found the call-back service unfavourable in conjunction with their lives. Women expressed a general dissatisfaction with Quitline and felt that information on cessation in pregnancy had not been updated over time. Women further reflected that they found the Quitline process an impersonal one and reminiscent of the ‘conveyor belt’ experience in antenatal care.

Of particular relevance here, is comparison of where the local South Australian approach to smoking cessation in pregnancy sits in the wider context of international best practice. The National Institute for Health and Care Excellence (NICE) provides up-to-date evidence-based recommendations on managing tobacco dependence in pregnancy. The guidelines indicate that brief interventions (similar to the 5A’s implemented as a minimum in SA) alone are unlikely to sustain smoking cessation in pregnancy and beyond [55]. The recommendation is for intensive ongoing support/intervention, including the use of continuous carbon monoxide (CO) monitoring, behavioural support and NRT use at the earliest opportunity in pregnancy [55, 56]. In the UK, a stop-smoking intervention entitled BabyClear increased cessation rates by 80% in pregnancy when implemented [57]. This program encouraged greater collaboration between government services, introduced universal CO monitoring at the first antenatal appointment with a routine opt-out referral for a positive reading and an explicit follow up protocol for smoking cessation services [57]. Furthermore, incentive schemes are promoted and encouraged as being effective for smoking cessation in pregnancy and health providers in the UK can refer women to such a scheme at their first antenatal appointment [55]. In Scotland and the USA, offering financial incentives for smoking cessation in pregnancy have shown higher cessation rates when compared to control groups [58–60]. While there will always be discrepancy between *best* practice guidelines and actual practice guidelines (as evident in the current study), the outdated SA guidelines clearly lag international best practice. It must be recognised that even if health providers in SA follow all initiatives in the current SA perinatal practice guidelines for smoking cessation in pregnancy, women are still at a disadvantage for successful smoking cessation. In the UK, specialised midwives and a ‘stop smoking in pregnancy service’ with the exclusive aim of working with women to improve smoking cessation rates in pregnancy have been introduced [57]. As the previous paragraph indicates, health providers already experience time restrictions, overwhelming workloads and work in systems that are not conducive for supporting implementation and monitoring of smoking cessation in pregnancy [39, 51, 61]. Women in SA will continue to be disengaged from smoking cessation if they do not have a supportive health service that utilises contemporary, evidence-based approaches and health providers that have time and capacity to implement them. Women interviewed here, also described lack of rapport with some health providers as a barrier to having difficult conversations about smoking behaviour. Home visiting antenatal health providers in Amsterdam affirm that continuity of care, and the ability to build a trusting relationship with women positively impacts their ability to have difficult conversations about smoking behaviour [39]. However, if smoking behaviour is discussed too early in the clinical relationship, prior to the development of trust, this can act as a barrier to smoking cessation [39]. Furthermore, by building rapport with women antenatally, health providers are able to better understand women’s needs and therefore practice woman centred care [39]. For women interviewed here and in Weiland et al. [40], woman centred care regardless of continuity, is a desirable antenatal care characteristic, along with wanting to feel valued and respected by health providers regardless of smoking status.

Due to the isolation, marginalisation and disempowerment women reported in their antenatal care, it is unsurprising that they want support from their peers who have similar experience. Women have described using prenatal classes inadvertently as a way of connecting with other women who smoke in pregnancy or who quit to develop peer support [40]. Currently, there is limited evidence as to the effectiveness of peer support for smoking cessation [62, 63]. What this finding may suggest however, is that women are seeking understanding and empathy from like-minded women because this need is not currently being met. Additionally, the need for personal agency and self-determination with regard to smoking cessation in pregnancy are also not being met. Women would like the opportunity to govern their own quit journey autonomously, with information and support from health providers.

It is noteworthy that women interviewed describe smoking in pregnancy as a ‘choice’ without consideration of the various influences on smoking behaviour, including physiological addiction. Women related having poor motivation or willpower when it came to smoking cessation in pregnancy and expressed that they were not ‘strong enough’ to quit. This sentiment has also been expressed by women anonymously in online forums in a Polish study [49]. The Australian public health messaging around smoking is designed to shock and shame people into quitting, and frames smoking as irrational and a failure of human control [64, 65]. However, this framing assumes that the rationalised body is dominated by the conscious will [65]. It is this discourse that represents continuation of smoking as a failure of self-control, self-discipline and weakness [65]. While this messaging is effective to encourage smoking cessation in some, it does not consider the self-esteem and self-efficacy or physiology impacting women who smoke in pregnancy [64, 65]. Further to this, it has been previously proposed that there are greater stressors for socioeconomically disadvantaged populations [66]. As such, individuals will seek the ‘feel good’ dopamine release that coincides with smoking tobacco, due to having less internal and/or external experiences capable of activating a dopamine release [44]. Additionally, the ‘risk taking’ behaviour of smoking may be the only opportunity for women to exert control in an otherwise uncontrollable existence [64, 66]. The public health messaging is therefore unhelpful and ultimately fuels the positive feedback loop of smoking and dopamine release. In order to assist smoking cessation in pregnancy, it may be more appropriate to consider the nested hierarchy approach which tracks the ‘streams of causation’ described by Glass and McAttee [67]. This model proposes that behaviour is influenced by ‘structured contingencies within the social and physical environment and by biological phenomena’ [67]. Therefore, for women to consider smoking cessation in pregnancy, a holistic approach is required that considers the physiology, psychology and sociology [67] of smoking. The current NICE guidelines are closer to this ideal when compared to the existing SA PPGs.

## Conclusion

In conclusion, four interconnected themes were identified to understand how women’s interaction with health providers can influence smoking cessation efforts antenatally. Social barriers to smoking cessation that exist because of the first theme ‘smoking embedded in women’s challenging lives and pregnancies’ are at the core of the other three themes. For women, interaction with the healthcare system contributes negatively to antenatal smoking cessation attempts with the themes ‘cyclic isolation and marginalisation’ and ‘feeling disempowered’ functioning as additional barriers. Using the ‘two sides of a coin’ analogy, it is the feelings of disempowerment that drive the strong desire women feel for ‘autonomy and self-determination’ in the final theme. This final theme represents the change women would like to see in healthcare. Women appeal for a greater understanding from health providers of the barriers they face with smoking cessation (physiological, psychological and sociological) and they would like to see this informing the South Australian public health approach antenatally to smoking cessation, as it does in international best practice. Also, they would like health practitioners to provide them with consistent information about risk and smoking cessation strategies for use in pregnancy so they can make informed choices given their personal circumstances. Improvements in SA antenatal care that increase understanding and information, as well as choice for women, consistent with international benchmarks, could lead to better woman centred care. Increasing resources and training, and allowing greater accessibility, may help women feel more support and encouragement to attempt cessation antenatally, rather than the isolation, marginalisation and disempowerment they currently feel.

## Supplementary Information

**Additional file 1.**

## Data Availability

The data generated during and/or analysed during the current study are not publicly available in accordance with ethical approval and participant consent for the study. Any bona fide researchers wanting to access data from this study would be contingent on further approvals from the Central Adelaide Local Health Network (CALHN) Human Research Ethics Committee (HREC). Researchers should contact Prof Lisa Smithers.
