# Supplementary material for: Isolation, marginalisation and disempowerment – understanding how interactions with health providers can influence smoking cessation in pregnancy
Source: BMC Pregnancy Childbirth. 2022 May 10;22:396. doi: 10.1186/s12884-022-04720-0 (PMC9086664; doi:10.1186/s12884-022-04720-0)
Supplement: Supplementary file 1 — Additional file 1. [file 12884_2022_4720_MOESM1_ESM.docx]

**APPENDICES**

Additional file 1 – Interview Schedule for Women Attending Focus Groups or Interviews

- Women’s experience, reflection and emotion around smoking in pregnancy.
- Women’s autonomy and what drives their desire to smoke.
- Women’s knowledge and understanding of risk associated with smoking in pregnancy.
- Antenatal conversations about smoking with health professionals.
- Discussion about Quitline being the only choice offered to women for quitting antenatally.
- Discussion about alternative antenatal support & methods for quitting smoking in pregnancy.
